# First- and Second-Generation Migrants: Attitudes Towards Homosexuality: The Role of Generation, Gender, and Religion

**DOI:** 10.3390/bs15091190

**Published:** 2025-08-31

**Authors:** Gaetano Di Napoli, Maria Garro, Marco Andrea Piombo, Cinzia Novara

**Affiliations:** Department of Psychology, Educational Science and Human Movement, University of Palermo, 90100 Palermo, Italy; gaetano.dinapoli@unipa.it (G.D.N.); maria.garro@unipa.it (M.G.); marcoandrea.piombo@unipa.it (M.A.P.)

**Keywords:** dual stigma, homonegativity, homosexuality, migrants, resilience, well-being

## Abstract

In Italy, the debate on migrants often focuses on issues such as social integration, economic conditions, and access to services. However, a little-investigated aspect concerns the double stigmatization of LGBTQ+ migrants, a reality made invisible by both the lack of research and the strict anti-LGBTQ+ laws present in many countries of origin. This study aimed to compare homonegativity levels between first- and second-generation migrants. A quantitative approach was used, with 127 participants (age 18–55, M = 30.63, SD = 11.58) completing an anonymous online questionnaire in three different languages. The instrument included a sociodemographic form and the Italian Scale for Measuring Homonegativity. A multivariate General Linear Model (GLM) analysis revealed significant effects of generation (*p* < 0.001, η^2^ = 0.688) and gender (*p* < 0.01, η^2^ = 0.144), with second-generation youth and women reporting lower levels of homonegativity. Religious affiliation had a minimal impact, influencing only the perception of deviance towards gay men (*p* < 0.05). Interactions between factors were generally non-significant, except for gender and religion. These findings underline the importance of generational and gender differences in the formation of homonegativity and highlight the need for further research to explore the cultural and social dynamics influencing these attitudes. In addition, there is a need to further explore how experiences of discrimination influence the well-being of LGBTQ+ migrants and what resilience strategies are adopted to address the challenges of homonegativity and marginalization.

## 1. Introduction

In contemporary times, the challenges faced by ethnic and sexual minorities have become central and urgent. Community psychology offers a crucial approach to promoting well-being and resilience by valuing the active role of both host and migrant communities in the processes of inclusion (Adger, 2000; Bonanno, 2004; Wilson, 2012).

Within this framework, the concepts of resilience and well-being assume a central role, not merely as individual outcomes but as collective processes that emerge through the interaction between individuals and their socio-ecological environments (Riemer et al., 2020). Resilience should not be understood as a static or innate trait but rather as a dynamic, situated, and relational capacity that enables individuals and groups to navigate adversity through adaptive strategies, everyday practices of resistance, and the construction of shared meanings (Ungar, 2011; Frost et al., 2016; Gatt et al., 2020; De Rose & Taddei, 2023).

Similarly, well-being cannot be reduced to a solely psychological or individual state; it involves a relational and social dimension that encompasses the public recognition of identities, access to affirming support networks, and engagement with culturally competent and inclusive institutions (García-Cid et al., 2020; Starks et al., 2023; Lattanner et al., 2024).

In the specific case of ethnic minorities, migration is characterized by the movement of individuals or groups from countries of origin to countries of destination—events and factors that inevitably affect the lives of individuals and the structures of the host societies (Van Hear et al., 2020). Sexual minorities include individuals or groups whose sexual identities deviate from normative heterosexuality and encompass, among others, lesbian, gay, bisexual, queer, and asexual people. These subjectivities face a range of interconnected challenges related to social recognition, cultural legitimacy, and access to rights—challenges that are often exacerbated by additional axes of marginalization, such as migratory status, ethnicity, or religious affiliation (Meyer, 2003; Zecena, 2019). However, it is essential to emphasize that the LGBTQ+ community is not a homogeneous entity but rather a diverse constellation of subjectivities with different lived experiences, social positions, and needs (Closson & Comeau, 2021). For instance, the experiences of transgender women differ significantly from those of bisexual men, whose presence is often marginalized or rendered invisible in public discourses. This invisibility may contribute to the persistence of stereotypes, limited social recognition, and exclusion from targeted services, thereby generating subtle yet persistent discrimination (Monaco, 2021; Jaspal et al., 2023).

A nuanced understanding of these internal differences is crucial for developing inclusive policies and interventions that can effectively respond to the specific needs of each segment of the LGBTQ+ population while avoiding reductive generalizations.

The intersection of ethnic and sexual minorities generates specific vulnerabilities and poses significant challenges for individuals who belong to both groups, requiring targeted attention and approaches that are sensitive to the multiple dimensions of identity. In this sense, community psychology offers a useful theoretical framework for understanding levels of homonegativity among heterosexual migrants, as it emphasizes the interaction between the individual and the social context in which they are embedded (Prilleltensky & Nelson, 2017). Homonegativity refers to “negative beliefs, emotions, and attitudes towards behaviors, identities, relationships, and individuals who are not heterosexual” (Herek, 2000, p. 19). This approach departs from individualistic perspectives and highlights the roles of social networks, institutions, and collective dynamics in shaping attitudes and beliefs (Williamson, 2000; Garro et al., 2022). Specifically, in the case of first-generation migrants and second-generation youth, community psychology allows us to analyze how belonging to a specific social network—be it familial, cultural, or religious—affects the construction of meanings related to sexual diversity (Di Napoli et al., 2025).

A central factor in this perspective is community resilience, which is understood as the capacity of a group to face change and foster positive adaptation processes (Norris et al., 2008). Resilience, far from being an individual trait, has a deeply social nature, as highlighted by its emphasis on relational dimensions (Cacioppo et al., 2011). Social resilience is defined as the collective ability to activate, maintain, and value positive relationships, which can serve as fundamental resources for facing and overcoming shared experiences of adversity. According to the most recent developments in the literature, the value of this construct lies precisely in the importance attributed to emotional and relational aspects, which are considered central to promoting adaptive responses and activating collective actions capable of generating cohesion and social transformation (Wilson, 2012). In the context of migration, this concept becomes particularly relevant, as ties to the community of origin and integration with the host society can either reinforce conservative attitudes or promote new forms of openness.

In this framework, homonegativity cannot be considered a simple individual trait but must be understood as a phenomenon constructed and maintained within community dynamics (Herek, 2000; Bogaert & Hafer, 2009). Cultural norms transmitted through family and social networks influence how sexual diversity is perceived, and first-generation heterosexual migrants often retain traditional values learned in their countries of origin (Morrison et al., 2009). However, second-generation youth in Western countries (in this case, referring to minors born in their parents’ country of destination) grow up from childhood in a social environment characterized by more inclusive values. Therefore, they may develop a more open and accepting perspective than the first generation, even though they may still face identity tensions related to cultural belonging (Lingiardi, 2011; Giunti et al., 2019; Strano et al., 2022).

The change in attitudes is not an exclusively individual process but depends on the opportunities offered by the intergenerational environment and the ability of communities to promote intercultural dialogue. Therefore, homonegativity in migrant communities should be analyzed within this network of influences, considering how intergenerational and intercommunity interactions can act as barriers or facilitators in the construction of a more inclusive view of sexual diversity (Farooqui & Kaushik, 2022).

In Italy, migration is characterized by the increasing diversification of migrant profiles, including a significant presence of LGBTQ+ individuals. Although precise official data on queer migration are lacking, estimates indicate a steady rise in this population, which remains largely underrepresented in national statistics (ISTAT, 2023). The Italian sociopolitical context is complex and multifaceted, shaped by a deeply rooted cultural and religious tradition that tends to be more conservative than those of other European countries (Abbatecola et al., 2022). Institutionally, reception and integration policies exhibit notable gaps and territorial disparities in their implementation. Major urban centers such as Rome, Milan, Palermo, and Naples provide more structured support networks and dedicated services, including specific programs for LGBTQ+ migrants run by NGOs and local organizations (Arcigay, 2022). Conversely, peripheral and rural areas face a scarcity of specialized services and smaller LGBTQ+ communities, which exacerbates isolation and vulnerability for these individuals (ISTAT, 2022). Furthermore, the Catholic Church’s predominant influence significantly shapes social attitudes and public policies, fostering a conservative climate that limits visibility and legal protections for LGBTQ+ persons. Migrants face the dual challenge of navigating both the host country’s norms and the traditionalist values of their communities of origin. Despite legislation prohibiting workplace discrimination based on sexual orientation (Legge n. 215/2021, 2021), these protections are frequently inadequate and poorly enforced in sectors such as education, healthcare, and public services, where LGBTQ+ migrants remain particularly at risk of exclusion and marginalization (FRA, 2021; Callahan & Loscocco, 2023).

This institutional and social fragmentation creates an environment in which LGBTQ+ migrants encounter discrimination and barriers to essential services, resulting in their heightened vulnerability. Targeted and integrated interventions, especially in underserved peripheral areas, are urgently required. Enhanced awareness and coordinated action among public institutions, civil society organizations, and migrant communities are crucial to promote inclusion and safeguard LGBTQ+ migrants’ rights in Italy.

Homosexual migrants may experience severe stress that compromises their well-being and quality of life. Additionally, they may suffer from strong marginalization and social exclusion, as well as violence perpetrated in the host countries—not only from the host community but also from their own ethnic community that has settled in the destination country—thus facing what is known as double stigma (M. Colombo et al., 2022).

Scientific research on homosexual migrants is scarce and typically focuses on language erosion in adults following the migration process (Wong Fillmore, 1991) or on the difficulties of learning and acquiring a second language (L2) (Cummins, 2000; Liddicoat & Taylor-Leech, 2014; Di Napoli et al., 2024). Moreover, there is a noticeable lack of research focusing on sexual orientation and gender identity among allogenous youth, likely because in many of their countries of origin, homosexuality is criminalized through laws that include penalties based on sexual orientation, including imprisonment, corporal punishment, and even the death penalty (Alessi et al., 2016; Hopkinson, 2017; Zecena, 2019). As of today, homosexuality is criminalized in 69 countries worldwide, half of which are located in Africa. For instance, in Mauritania, Somalia, and northern Nigeria, homosexual acts are punishable by death (ILGA, 2024).

In Italy, the international protection system safeguards homosexual migrants who flee their countries of origin because of their sexual orientation or gender identity. Article 10 of Council Directive 2004/83/EC of 29 April 2004 (Official Journal of the European Union, 2004), recognizes sexual orientation and gender identity as distinctive characteristics of a person and, thus, grounds for international protection.

Migrant communities and informal migrant networks cannot always be considered safe spaces where homosexual migrants can develop forms of solidarity and support essential in the host country, as the double stigmatization to which they are exposed adds further difficulties for them.

## 2. The Present Study

This study explores how experiences and perceptions of homonegativity manifest across different generations of migrants, focusing on three key variables: gender, generation, and religion. These dimensions are considered potential predictors of homonegativity and are particularly relevant for analyzing intra-community dynamics (Grimbos et al., 2010; Herek & McLemore, 2013; Van den Akker et al., 2013). In particular, gender can significantly influence such attitudes, highlighting differences between men and women in terms of acceptance or rejection of homosexuality (Collier et al., 2013; De la Torre-Pérez et al., 2022; Herek, 2002). Meanwhile, religion, through its doctrinal and moral prescriptions, can contribute to the formation of heteronormative or discriminatory beliefs and attitudes (A. D. Colombo & Barbagli, 2007). The generational variable, on the other hand, is crucial for understanding the evolution of social representations of homosexuality among first- and second-generation migrants: new generations, often more exposed to the values of the host society and to a more inclusive educational context, may show less rigid attitudes than previous generations (Bourhis et al., 1997; Diaz et al., 2001). However, this openness is not automatic, as it may conflict with norms transmitted by families of origin or ethnic communities, generating identity tensions and value-based ambivalence. The joint examination of these variables allows for a more comprehensive and in-depth understanding of the manifestations of homonegativity within heterosexual migrant communities.

The general objective of this research is to detect attitudes toward homosexuality in a group of first-generation heterosexual migrants and second-generation youth currently living in Southern Italy. The research hypotheses are as follows:

- Hypothesis 1: first-generation heterosexual migrants exhibit higher levels of homonegativity than do second-generation youth.
- Hypothesis 2: men show higher levels of homonegativity than women do.
- Hypothesis 3: Muslim migrants exhibit higher levels of homonegativity than Christian migrants.

The adopted methodology is based on a quantitative approach that allowed for the measurement of the intensity of attitudes toward homosexuality and homosexual individuals, as well as the performance of inferential statistical analyses useful for testing specific hypotheses regarding differences and associations among the variables examined.

## 3. Materials and Methods

### 3.1. Participants and Procedures

A total of 127 migrants of various nationalities residing in Palermo participated in this study. Specifically, the sample included 62 first-generation migrants (M = 40.5; SD = 8.40) and 65 second-generation youth (M = 21.23; SD = 3.61), with an age range of 18–55 years (Table 1).

Participants were recruited using a snowball sampling method, which allowed the researchers to reach and engage a network of individuals belonging to the target population. This was achieved with the support of third-sector organizations operating in the area and through an open invitation published on major social media platforms, providing a direct link to access the questionnaire.

The objectives and voluntary nature of this study were translated for participants into several languages (Italian, English, and French), ensuring that all participants could clearly understand the purpose of this study. Informed consent was obtained, which included details about privacy and data handling and information on the anonymization procedures of the results. Additionally, to ensure informed participation, an information sheet was provided that clearly explained the participants’ rights, including the option to withdraw from the study at any time without consequences.

This project was approved by the Ethics Committee for Psychological Research of the University of Palermo (protocol n. 49/2021), which evaluated this study’s compliance with ethical standards.

The data collection procedure was fully compliant with the Code of Ethics for Research of the Italian Association of Psychology and the ethical guidelines of the Declaration of Helsinki, as well as the standards of the American Psychological Association (APA) regarding participant treatment.

### 3.2. Measures

The anonymous online questionnaire, available in three languages (Italian, English, and French), was divided into two sections. The first part consisted of a socio-demographic form used to collect the following information: age, generation, gender, religion, educational level, employment status, relationship status, presence and age of children, religious affiliation, living arrangements, country of birth, and length of residence in Italy. The second part included the Italian Scale for Measuring Homonegativity (SIMO) (Lingiardi & D’Augelli, 2005), which is described in detail below.

### 3.3. Homonegativity

Specifically, the first version of the SIMO questionnaire was used, consisting of 56 items on which participants were asked to express their degree of agreement or disagreement using a 5-point Likert scale (1 = totally disagree, 2 = disagree, 3 = I do not know, 4 = agree, 5 = totally agree). The 56 items were divided into two sections: 28 items assessing attitudes toward gay men and 28 items assessing attitudes toward lesbians. When the respondent is male, the scale first presents items related to attitudes toward gay men, followed by those toward lesbians; for female respondents, the order is reversed.

The scale is divided into three subscales, each designed to explore a specific dimension of homonegativity:

- Deviance measures the extent to which homosexuality is perceived as pathological, immoral, or abnormal (e.g., “gays can be straight if they want to”; “being gay is a psychological disorder”; “male homosexuality poses a threat to the family as a value and a social institution”).
- Rights evaluate attitudes toward the civil rights of homosexual people (e.g., “even if a male political candidate publicly declares himself gay, I would vote for him”; “I think gay marriages should be allowed by the law”; “cinema, television, and newspapers provide an image that is too favourable for male homosexuality”).
- Socialization explores discomfort or resistance regarding close social interactions with homosexual individuals (e.g., “if a friend confides to me that he is gay, I think our friendship would be compromised”; “seeing a couple of men with a romantic attitude bothers me”; “working with a lesbian colleague makes me uncomfortable”).

A total score ranging from 56 to 280 was obtained by summing the individual item scores. A higher SIMO score corresponds to a higher level of homonegativity.

Finally, the instrument was administered in its original Italian version for second-generation participants, while first-generation participants were given the option to choose between the English and French versions to ensure better comprehension of the questions.

### 3.4. Statistical Analysis

Statistical analyses were performed using SPSS software (version 24). Descriptive statistics were calculated for all relevant variables to provide an overview of the data distribution and identify any outliers or issues related to normality.

A multivariate General Linear Model (GLM) was used to examine the impact of three main variables—generation, gender, and religion—on a set of dependent variables related to homonegativity attitudes toward gay men and lesbians. This approach was chosen for its ability to analyze multiple dependent variables simultaneously, preserving the multivariate structure of the questionnaire, and minimizing the risk of Type I errors resulting from separate analyses. Generation and religion were treated as fixed factors for each dimension of the SIMO questionnaire, whereas sex was treated as a dichotomous variable (M = 1, F = 2).

The model included all scores from the three subdimensions of the SIMO questionnaire: deviance, rights, and socialization, each referring separately to attitudes toward gay men and lesbians as dependent variables. The decision to analyze gay and lesbian factors separately allowed for the exploration of specific differences between the two groups that may emerge in relation to the independent variables.

To further investigate these influences and determine which demographic groups presented significant differences, a series of between-subject effects analyses was conducted for each dependent variable. Subsequently, specific contrasts were created to directly compare particular groups within each demographic category (e.g., different generations and religious affiliations). These contrasts were helpful in identifying which subgroups contributed most to the differences observed in the overall model.

The significance level was set at α = 0.05 for all analyses to ensure proper control of the risk of false positives. However, effect size values, such as partial eta-squared (η^2^), were calculated to estimate the proportion of variance explained by significant effects. This provided a measure of the practical relevance of the results, beyond mere statistical significance.

## 4. Results

### 4.1. Overall Multivariate Test Significance

A multivariate General Linear Model (GLM) was used to assess the impact of generation, religious affiliation, and gender on a series of dependent variables related to attitudes toward gay men and lesbians.

The multivariate analysis revealed a significant main effect of generation on all measured dimensions of homonegativity (Wilks’ Lambda = 0.312, F(6, 114) = 41.873, *p* < 0.001, partial η^2^ = 0.688). This result indicates that generational differences account for approximately 68.8% of the variance in attitudes, suggesting a substantial shift in attitudes across generations. The findings reflect a trend in which younger generations tend to exhibit more positive and open attitudes toward the rights of gay men and lesbians than older generations, consistent with previous studies highlighting greater social acceptance among youth (Baunach, 2012; Twenge et al., 2016; Hadler & Symons, 2018).

A significant main effect was also found for gender (Wilks’ Lambda = 0.856, F(6, 114) = 3.201, *p* < 0.01, partial η^2^ = 0.144). This result shows that gender moderately contributes to the variance in attitudes, explaining approximately 14.4% of the total variance in attitudes. Consistent with previous research, women tend to hold more positive attitudes toward the rights of gay and lesbian individuals than men, suggesting greater sensitivity to social and inclusion-related issues (Herek, 2002; Breen & Karpinski, 2013).

In contrast, religious affiliation did not show a significant multivariate effect (Wilks’ Lambda = 0.901, F(6, 114) = 2.089, *p* > 0.05, partial η^2^ = 0.099). Although religious affiliation contributed only minimally to the variance in scores (approximately 9.9%), this result may indicate that differences related to religion, while present, are not pronounced enough to emerge in the overall multivariate model.

A significant interaction effect was found between religion and gender (*p* < 0.05). This suggests that differences in attitudes toward the rights of gay men and lesbians are particularly pronounced for specific gender groups within religious categories. Women belonging to religious groups tended to express less negative attitudes than men in the same religious categories, indicating a complex dynamic between religious values and gender sensitivity.

In contrast, no significant interaction effects were found for the following:

- Between generation and religion.
- Between generation and gender.
- The three-way interaction among generation, religion, and gender (all *p* > 0.05).

These results indicate that while generation and gender exert significant and independent main effects, their combined influence with religious affiliation does not produce significant interaction effects. Therefore, the observed differences in the data are mainly attributable to the individual effects of the independent variables rather than their combinations.

Overall, these findings highlight generation as the most impactful factor, accounting for a considerable portion of the variance and confirming the central role of generational dynamics in shaping social attitudes toward the elderly. Although religious affiliation did not show a significant main effect, its interaction with gender suggests a more complex influence that warrants further analysis within specific subgroups of the population. Finally, the contribution of gender underscores the persistence of disparities in social perceptions between men and women, reflecting potential differences in value systems and social sensitivity toward the rights of gay and lesbian individuals (Table 2).

### 4.2. Between-Subject Effect: Mean Differences in Homonegativity Attitude Scores Between First and Second Generation of Migrants

Univariate tests explored in detail the effects of generation on homonegativity attitudes toward gay men and lesbians, revealing significant effects across all measured dimensions of homonegativity (all *p* < 0.001) (Table 3). Post hoc analyses using contrast matrices highlighted a consistent generational gap. Specifically, regarding gay rights, first-generation migrants reported significantly higher levels of homonegativity than second-generation participants, suggesting a greater resistance among first-generation migrants to accepting gay rights. This gap may reflect deeply rooted cultural and social values from their countries of origin, which influence their attitudes toward civil rights in host societies.

The same pattern was observed for the deviance and socialization subdimensions related to gay men, with first-generation migrants again showing significantly higher levels of homonegativity than their later-generation counterparts. These findings suggest that socialization experiences and cultural influences in the country of origin may shape negative perceptions of sexual minorities in the host country.

Regarding attitudes toward lesbians, the results mirrored those for gay men, with first-generation participants perceiving lesbian rights more negatively. The perception of deviance, another key indicator of homonegativity, also showed significantly higher scores among first-generation migrants. Additionally, first-generation members showed a significantly lower willingness to socialize with lesbians. These findings reinforce the idea that the integration of lesbian individuals into host societies is perceived more negatively by those from more conservative cultural backgrounds.

Regarding religion, univariate tests showed a significant effect only on deviance scores toward gay men (F = 6.25, *p* < 0.05), while no significant differences emerged in other attitudes. Post hoc analyses using contrast matrices revealed that Muslim participants perceived gay men as more deviant than Christian participants, suggesting that religious affiliation significantly influences perceptions of deviance in gay individuals. A similar but non-significant trend was observed in deviance scores toward lesbians. These findings may reflect the influence of moral and cultural values specific to each religion, especially regarding viewing homosexuality as deviant behavior (Wilcox, 2003).

However, the limited effect of religious affiliation on other attitudes may indicate that the migratory context and growing secularization in host societies play a key role in reshaping traditional religious views toward sexual minorities.

Univariate tests indicated that gender had a significant effect only on perceived deviance toward gay men (F = 5.29, *p* < 0.05, partial η^2^ = 0.04) and lesbians (F = 4.44, *p* < 0.05, partial η^2^ = 0.03). The gender effects on gay rights (F = 0.84, *p* > 0.05, partial η^2^ = 0.00), lesbian rights (F = 0.48, *p* > 0.05, partial η^2^ = 0.00), and socialization with lesbians (F = 0.00, *p* > 0.05, partial η^2^ = 0.00) were not statistically significant. Post hoc analyses with contrast matrices showed that men tended to hold more negative attitudes than women, especially regarding the perception of deviance attributed to gay and lesbian individuals. These results suggest that gender differences are more pronounced in value- and morality-based dimensions, such as deviance, than in the recognition of rights or willingness to socialize.

This finding aligns with previous literature, which suggests that traditional masculinity norms may lead men to stigmatize sexual behaviors that deviate from conventional gender roles. Consequently, men might exhibit greater resistance in recognizing the legitimacy and acceptability of sexual minorities, particularly concerning behaviors perceived as challenging traditional conceptions of masculinity (Herek, 1986; Sánchez et al., 2010; Pascoe, 2011).

The analysis of interaction effects revealed a significant interaction between generation and gender on deviance scores attributed to gay men, suggesting that younger or older generations may express different levels of homonegativity depending on their gender. This effect may reflect varying socialization experiences based on both gender and age, with younger-generation males potentially displaying more tolerant attitudes, while older-generation males may be more resistant to change.

The three-way interaction between generation, religious affiliation, and gender had a significant impact on lesbian rights (F = 4.19, *p* < 0.05, partial η^2^ = 0.03), indicating that religious and gender experiences influenced the perception of lesbian rights in different ways depending on the generation.

However, other interactions among variables did not show significant effects, suggesting that the main effects of individual variables (generation, gender, and religion) are more influential than their combined effects, particularly regarding homonegativity.

Overall, these findings highlight the decisive role of generation, gender, and religion in shaping homonegative attitudes toward LGBTQ+ individuals. Generational differences emerged as the most significant factor, suggesting that younger generations are more inclined to adopt tolerant attitudes toward sexual minorities than older generations. This may be due to socialization in more inclusive environments and greater exposure to social movements, awareness campaigns, and cultural shifts that promote civil rights and equality. Moreover, younger generations grow up in a social context increasingly characterized by the recognition of diversity and the rights of sexual minorities, which may positively influence their attitudes.

In line with Berry’s (1997) acculturation hypothesis, prolonged exposure to inclusive values within the host society—through social media and formal education—may foster greater openness and tolerance toward gay and lesbian individuals and, more broadly, toward the LGBTQ+ community. The significant gender × religion interaction observed in our data reveals a more differentiated pattern. Within both the Muslim and the Christian sub-samples, men reported markedly higher deviance scores than women of the same affiliation. In contrast, gender differences were negligible among participants who declared no active religious practice. Table 3 summarizes the univariate between-subject effects (F-values, partial η^2^, observed power) of generation, gender, religious affiliation, and their interactions on each of the six SIMO sub-dimensions.

## 5. Discussion

In general, the results confirmed Hypothesis 1, namely that first-generation heterosexual migrants exhibited significantly higher levels of homonegativity than second-generation youth. This finding is consistent with previous studies comparing first- and second-generation migrants (Kalmijn & Kraaykamp, 2018; Röder, 2015). These studies show that second-generation youth tend to have more tolerant attitudes, likely influenced by socialization in a more inclusive environment and the growing visibility of the LGBTQ+ community.

In this sense, young people may play a significant role in promoting change within their families and communities. The more open and inclusive education to which they are exposed—one that values diversity and acknowledges the rights of LGBTQ+ individuals—may facilitate the renegotiation of their traditional beliefs. Through intergenerational dialogue with parents and other community members, younger generations may help drive a gradual transformation of the social representation of homosexuality, thus bridging the generational gap in terms of acceptance and inclusion.

With regard to Hypothesis 2, the results showed that men expressed higher levels of homonegativity than women. Although this cannot be generalized to all women, some cultural and social elements may help explain this difference, such as greater exposure to inclusive educational messages or a socialization process that promotes empathy and sensitivity to social issues. It has also been hypothesized that men may perceive male homosexuality as a symbolic threat to traditional masculinity, which is often based on rigid normative models. This resistance may reflect a deeper internalization of heteronormative and homophobic norms among men, leading to more negative evaluations of gay individuals.

Hypothesis 3 was partially confirmed, as a significant effect was found on the “deviance” subscale for gay men. In particular, Muslim participants were more likely to associate male homosexuality with deviant behavior. In many Muslim-majority countries, homosexuality is subject to strong stigmatization or outright condemnation, and such attitudes may also shape the views of migrant individuals. The Muslim religion tends to be more conservative toward sexual minorities (Habib, 2009). This finding is further supported by Röder (2015), who highlighted that Muslim and Eastern Orthodox migrants tend to hold less accepting attitudes toward homosexuality than Catholics and Protestants. Such resistance or disapproval, particularly among first-generation Muslim participants, was also evident in the present study. Specifically, 20 first-generation Muslim participants refused to complete the questionnaires. This reluctance to participate in the research may be interpreted as a form of rejection toward topics perceived as conflicting with their religious beliefs and traditional practices or possibly as a result of personal discomfort due to a non-heterosexual orientation. Although the collected data offer meaningful insights, unexplored areas remain, especially regarding the evolution of attitudes across generations of migrants and the ongoing influence of religion and culture in shaping opinions on homosexuality.

Furthermore, the results underscore the importance of generation as the main determinant of attitudes toward gay and lesbian individuals among migrants. The significant difference between first- and second-generation participants suggests that young people growing up in more inclusive social environments tend to assimilate cultural norms that promote greater acceptance and equality.

Gender also emerged as a relevant factor, although with a more moderate impact than generation. Women generally expressed more positive attitudes toward homosexual individuals than men, especially regarding the rights of gay men and lesbians. However, a notable result concerned attitudes toward lesbians, where a reverse trend was observed: women reported slightly higher homonegativity than men. This phenomenon may reflect the persistence of cultural stereotypes linked to gender roles and social expectations regarding female relationships.

Finally, regarding religion, Muslim participants, particularly those from the first generation, showed higher levels of homonegativity than Christians. This suggests that religious affiliation continues to play a role in shaping attitudes toward homosexuality, although it did not emerge as a strong predictor in the multivariate model used in this study. However, the interplay between religion and gender reveals nuanced dynamics. Within specific religious groups, attitudinal differences may be shaped by various factors, including the degree of religious observance, the context in which individuals are socialized, and the influence exerted by communities and social networks.

## 6. Limitations and Future Direction

Among the limitations of the present study, it is important to first consider the small sample size (*N* = 127), which does not allow for a strong generalization of the findings to the entire migrant population living in Southern Italy. The sample included first- and second-generation heterosexual migrants of both Christian and Muslim faiths, but an equal representation among the various subgroups could not be ensured, which may have affected the comparability between categories.

Moreover, the research design was cross-sectional, which limits the ability to analyze the evolution of homonegative attitudes over time and prevents any causal conclusions about the relationships among the investigated variables. Data were collected through the online administration of the SIMO questionnaire via a link distributed by third-sector organizations operating in the region. This approach made it possible to reach a population that is often difficult to access, but also introduced potential bias due to the snowball sampling method. Although effective in migrant contexts, this technique tends to favor the participation of individuals belonging to relatively open social networks or those who are already sensitive to LGBTQ+ issues.

The exclusive use of a self-administered questionnaire also carries the risk of social desirability bias, especially given the sensitive nature of the topic. Future research may benefit from longitudinal designs, the inclusion of more diverse samples, and the consideration of a broader range of variables to deepen the understanding of the complex interrelations that characterize these phenomena.

This study may serve as an important foundation for future research seeking to further explore the individual and sociocultural variables influencing attitudes toward sexual minorities. Therefore, it is hoped that this work can offer a significant contribution not only to academic literature but also to social practice. Understanding the experiences of social minorities and acknowledging their challenges are essential steps in creating effective interventions that promote inclusion and respect for human rights.

## 7. Conclusions

The results showed that first-generation heterosexual migrants generally exhibit higher levels of homonegativity than second-generation youth. This trend fits within a broader framework concerning the transmission of attitudes toward homosexuality, which evolved from the first to the second generation. In this sense, second-generation youth tend to hold more tolerant attitudes toward sexual minorities, likely because they are socialized in a more inclusive and open environment than their parents. Consequently, while still influenced by the traditions and religious beliefs of their parents, second-generation youth may develop more tolerant and inclusive opinions due to their exposure to a more pluralistic society and liberal behavioral norms. The process of acculturation and openness toward sexual and identity diversity among second-generation youth represents a continuous dynamic of change—not only in terms of attitudes and values but also in terms of individual and collective experiences that are inevitably shaped by an increasingly inclusive and pluralistic environment (Marks & Conn, 2012; Coll & Marks, 2012).

Second-generation migrants grow up immersed in a society that—despite its contradictions—has made significant strides toward the inclusion of LGBTQ+ individuals through legislation, media visibility, and the promotion of values related to human rights and equality (Berggren et al., 2019; Craig et al., 2021). In many Western countries, including Italy, there has been a gradual shift in social attitudes toward homosexuality, with growing recognition of civil rights for LGBTQ+ people, such as same-sex marriage and anti-discrimination policies in Italy. True inclusion is not a static goal but a continuous process that requires commitment, listening, collaboration, and patience (Blanc, 2021).

Moreover, the increasing visibility of the LGBTQ+ community in the media and popular culture has helped raise awareness among younger generations, who are more exposed to themes of sexual and gender diversity. Films, TV series, social activism, and social media have played a crucial role in giving voice to the stories of gay, lesbian, bisexual, transgender, and nonbinary individuals, bringing issues of inclusivity and LGBTQ+ rights to the forefront of public debate. Younger generations, who constantly engage with this content, are more likely to adopt open attitudes toward sexual and gender diversity as they grow up in environments that promote understanding and acceptance of differences.

The findings suggest that although progress is being made in improving attitudes toward sexual minorities, significant obstacles persist, particularly those related to cultural, religious, and generational differences.

In light of these findings, it is equally important to consider the practical implications of the results. Understanding the intersectional dynamics that shape attitudes toward sexual and cultural minorities—particularly the interplay between generations, religions, and ethnic backgrounds—can offer valuable insights for designing more inclusive and effective educational, social, and political interventions.

Targeted interventions in schools, for example, could work to deconstruct gender and sexual orientation stereotypes through an intercultural approach that promotes meaningful interactions between individuals from diverse cultural, gender, and sexual identity backgrounds and recognizes these encounters as valuable opportunities for growth and inclusion.

At the institutional level, the findings suggest a need for participatory policies that actively involve both migrant and LGBTQ+ communities in setting priorities, ensuring that inclusion efforts are not imposed from above but rather co-constructed with—and not only for—the people they are intended to support. The third sector may also draw useful insights to promote projects that foster intergenerational dialogue, self-narration, and collective empowerment, with particular attention to the role of second-generation individuals as bridges between worlds, cultures, and perspectives.

Only through an approach capable of holding together structural and subjective dimensions, public policies, and everyday experiences will it be possible to envision and build a truly pluralistic, fair, and inclusive society.

Although this study focuses on a specific national and cultural context, the dynamics of exclusion and marginalization experienced by LGBTQ+ migrants must be situated within a broader macrosociological framework. The intersection of religious conservatism, migration, and sexual diversity reveals transnational patterns of double exclusion that are not confined to the Italian context. In particular, parallels can be drawn with the Israeli socio-political landscape, where a significant presence of migrants coexists with deep-rooted tensions between tradition and modernity.

In this regard, recent ethnographic studies by Ben-Lulu (2021, 2022) offer critical insights into how gender and queer identities are negotiated in religious spaces and how rituals can serve as forms of resistance or introspective re-signification. These contributions highlight how religious narratives and collective identities can simultaneously marginalize and create space for counter-narratives of belonging to emerge.

Integrating such comparative perspectives allows for a more comprehensive understanding of the structural mechanisms of exclusion and emphasizes the importance of critically positioning local analyses within global discourses on queerness, migration, and community dynamics in the Netherlands. The experiences of LGBTQ+ migrants cannot be fully understood in isolation from the broader global context and power structures that profoundly shape their trajectories. These individuals are situated at the intersection of multiple normative systems—cultural, religious, and institutional—that operate locally and transnationally. Their marginalization is not solely a product of Italian social dynamics but is embedded in wider processes involving the global circulation of norms regarding identity, sexuality, and belonging.

In this regard, the present study adopts an intersectional and multilevel perspective that links everyday lived experiences (micro-level) with systemic structures of exclusion (macro-level), thereby providing a more nuanced and politically informed analysis.

Integrating such perspectives highlights how LGBTQ+ migrant subjectivities are deeply shaped by intersectional positionalities that cannot be comprehended without a theoretical framework attentive to the transnational dynamics of power, alliances, and forms of symbolic and social resilience.

In conclusion, this study raises several questions about the meaning of inclusion and the role each of us can play in promoting it. It is clear that the challenges related to the dual vulnerability of social minorities cannot —and must not—be addressed with simplistic solutions or generalizations. Instead, they require an approach that is attentive to and respectful of their differences.

## Figures and Tables

**Table 1:** Socio-demographic information.

Variable	Category	*N* = 127 (%)
Gender	Man	60 (47.2%)
	Woman	67 (52.8%)
Generation	First	62 (48.8%)
	Second	65 (51.2%)
Religion	Christinaity	81 (63.8%)
	Islam	46 (39.1%)
Country of Origin	Ivory Coast	13 (12.4%)
	Ghana	41 (39.1%)
	Morocco	14 (13.3%)
	Mauritius	9 (8.6%)
	Nigeria	22 (21%)
	Senegal	9 (8.6%)
	Tunisia	9 (8.6%)
	Other	18 (14.2%)
Education	No Formal Education	3 (2.4%)
	Elementary School Diploma	6 (4.7%)
	Middle School Diploma	18 (14.2)
	High School Diploma	91 (71.7)
	Bachelor’s Degree	6 (4.7%)
	Other	3 (2.4%)
Employment Status	Employed	90 (70.9%)
	Unemployed	11 (8.7%)
	Self-employed	21 (16.5%)
	Retired	5 (3.9%)
Relationship Status	Not in a relationship	81 (63.8%)
	In a relationship	46 (36.2%)
Living Arrangements	Living alone	94 (74%)
	Living with partner	32 (25.2%)
Marital Status	Not married	78 (61.4%)
	Married	49 (36.2%)
Children	No children	81 (63.8%)
	Have children	46 (36.2%)
Years in Italy (first generation only)	1–5 years	6 (4.7%)
	6–10 years	11 (8.7%)
	11–15 years	15 (11.8%)
	More than 15 years	30 (23.6%)

**Table 2:** Multivariate overall results of the effect of generation, religion and gender on homonegativity attitudes towards gays and lesbians.

	Wilks’ Lambda	F	*p*-Value	η*p*^2^
Generation	0.31	41.87 ***	0.00	0.68
Religion	0.90	2.08	0.00	0.09
Gender	0.85	3.20 **	0.00	0.14
Generation * Rel.Aff.	0.92	1.60	0.15	0.07
Generation * Gender	0.93	1.41	0.21	0.07
Rel.Aff. * Gender	0.89	2.26 *	0.04	0.10
Generation * Rel.Aff. * Gender	0.90	1.98	0.07	0.09

* significance < 0.05; ** significance at <0.01; *** significance at <0.001.

**Table 3:** Between-subject effects of generation, gender, religious affiliation and their interaction on the six SIMO sub-dimensions.

		F	η*p*^2^	Observed Power
Corrected Model	Rights–Gay	35.75 ***	0.67	1.00
	Deviance–Gay	21.51 ***	0.55	1.00
	Socializing–Gay	29.76 ***	0.63	1.00
	Rights–Lesbian	47.82 ***	0.73	1.00
	Deviance–Lesbian	16.91 ***	0.49	1.00
	Socializing–Lesbian	26.96 ***	0.61	1.00
Generation	Rights–Gay	181.32 ***	0.60	1.00
	Deviance–Gay	82.31 ***	0.40	1.00
	Socializing–Gay	145.21 ***	0.55	1.00
	Rights–Lesbian	252.85 ***	0.68	1.00
	Deviance–Lesbian	67.61 ***	0.36	1.00
	Socializing–Lesbian	114.75 ***	0.49	1.00
Religious Affiliation	Rights–Gay	0.31	0.00	0.08
	Deviance–Gay	6.25 *	0.05	0.69
	Socializing–Gay	0.16	0.00	0.06
	Rights–Lesbian	0.00	0.00	0.05
	Deviance–Lesbian	3.23	0.02	0.43
	Socializing–Lesbian	3.05	0.02	0.41
Gender	Rights–Gay	0.84	0.00	0.14
	Deviance–Gay	5.29 *	0.04	0.62
	Socializing–Gay	3.47	0.02	0.45
	Rights–Lesbian	0.48	0.00	0.10
	Deviance–Lesbian	4.44 *	0.03	0.55
	Socializing–Lesbian	0.00	0.00	0.05
Generation * Rel.Aff.	Rights–Gay	1.14	0.01	0.18
	Deviance–Gay	0.54	0.00	0.11
	Socializing–Gay	2.30	0.01	0.32
	Rights–Lesbian	0.71	0.00	0.13
	Deviance–Lesbian	1.83	0.01	0.26
	Socializing–Lesbian	4.45 *	0.03	0.55
Generation * Gender	Rights–Gay	0.02	0.00	0.05
	Deviance–Gay	4.00 *	0.03	0.51
	Socializing–Gay	1.54	0.01	0.23
	Rights–Lesbian	0.21	0.00	0.07
	Deviance–Lesbian	2.05	0.01	0.29
	Socializing–Lesbian	2.30	0.01	0.32
Rel.Aff. * Gender	Rights–Gay	0.69	0.00	0.13
	Deviance–Gay	1.53	0.01	0.23
	Socializing–Gay	0.78	0.00	0.14
	Rights–Lesbian	1.16	0.01	0.18
	Deviance–Lesbian	0.24	0.00	0.07
	Socializing–Lesbian	1.07	0.00	0.17
Generation * Rel.Aff. * Gender	Rights–Gay	0.02	0.00	0.05
	Deviance–Gay	0.07	0.00	0.05
	Socializing–Gay	1.11	0.00	0.18
	Rights–Lesbian	4.19 *	0.03	0.52
	Deviance–Lesbian	0.45	0.00	0.10
	Socializing–Lesbian	1.01	0.00	0.17

* significance < 0.05; *** significance at <0.001.

## Data Availability

Data are available upon request due to privacy restrictions.
